# Mavacamten toxicity, hepatic dysfunction, and rifampicin: cause or coincidence?

**DOI:** 10.1093/ehjcr/ytag404

**Published:** 2026-06-02

**Authors:** Massimo Mapelli, Simona Costantino, Riccardo Maragna

**Affiliations:** Heart Failure Unit, Centro Cardiologico Monzino, IRCCS, University of Milan, Via Parea 4, Milan 20138, Italy; Heart Failure Unit, Centro Cardiologico Monzino, IRCCS, University of Milan, Via Parea 4, Milan 20138, Italy; Heart Failure Unit, Centro Cardiologico Monzino, IRCCS, University of Milan, Via Parea 4, Milan 20138, Italy

We read with great interest the recent case report by Lucas *et al*. describing severe cardiogenic shock attributed to mavacamten toxicity and successfully treated with rifampicin administration in a patient with obstructive hypertrophic cardiomyopathy.^1^ The authors should be congratulated for reporting an important and clinically relevant case highlighting the potentially life-threatening consequences of drug interactions during mavacamten therapy.

However, we believe that some aspects of the pathophysiological interpretation deserve further discussion.

First, although the reduction in plasma mavacamten concentration temporally followed rifampicin initiation, causality remains difficult to establish. Mavacamten is characterized by a prolonged and highly variable elimination half-life, ranging from approximately 9–23 days depending on metabolic status and clinical conditions.^2^ Therefore, the observed decline in plasma concentration after rifampicin administration may at least partially reflect the expected pharmacokinetic elimination of the drug after discontinuation, rather than a direct therapeutic effect of rifampicin itself. While rifampicin-mediated CYP induction is biologically plausible, the present report cannot definitively distinguish accelerated clearance from the natural washout phase of mavacamten accumulation.

Second, the causal role attributed to esomeprazole should probably be interpreted more cautiously. Although esomeprazole may inhibit CYP2C19 activity, it is generally considered a moderate rather than a strong inhibitor, with less pronounced effects on CYP3A4.^3,4^ It may be difficult to fully explain such a dramatic clinical presentation—including profound biventricular dysfunction and cardiogenic shock—solely on the basis of a short course of esomeprazole therapy. In our opinion, esomeprazole may have represented a contributing factor rather than the exclusive trigger of toxicity.

Third, we wonder whether acute hepatic dysfunction may have played a more central role in the development of mavacamten accumulation. The patient initially presented with severe gastrointestinal symptoms associated with marked hepatic cytolysis before the onset of overt cardiogenic shock. Since mavacamten is extensively metabolized by hepatic cytochromes, acute liver injury itself may have substantially impaired drug clearance and promoted progressive accumulation.^5,6^ In this scenario, hepatic dysfunction may have acted both as a precipitating factor and as part of a subsequent vicious circle involving negative inotropic drug exposure, worsening cardiac output, and further hepatic impairment.

Overall, we fully agree with the authors regarding the importance of careful monitoring for drug interactions and ventricular function during mavacamten therapy. Nevertheless, we believe that greater caution may be warranted before concluding that rifampicin administration was the only determinant of recovery or that esomeprazole alone represented the principal cause of toxicity. Importantly, rather than representing mutually exclusive hypotheses, several mechanisms may have contributed simultaneously to the patient’s clinical deterioration. A multifactorial process involving moderate CYP2C19 inhibition by esomeprazole, altered pharmacokinetics related to prior bariatric surgery, acute hepatic dysfunction with impaired drug metabolism, prolonged mavacamten half-life, and subsequent shock-related hepatic injury may together explain the observed toxicity. Likewise, the patient’s recovery may have reflected both progressive physiological drug elimination after discontinuation and a potential contribution of rifampicin-mediated enzymatic induction. In our opinion, framing this presentation as a complex differential diagnostic and pharmacokinetic scenario may provide additional educational value for clinicians managing patients treated with mavacamten.

## Data Availability

No new data were generated or analysed in support of this research.
